# Computer-Assisted Versus Manual Planning for Stereotactic Brain Biopsy: A Retrospective Comparative Pilot Study

**DOI:** 10.1093/ons/opz177

**Published:** 2019-08-05

**Authors:** Hani J Marcus, Vejay N Vakharia, Rachel Sparks, Roman Rodionov, Neil Kitchen, Andrew W McEvoy, Anna Miserocchi, Lewis Thorne, Sebastien Ourselin, John S Duncan

**Affiliations:** 1 Department of Neurosurgery, National Hospital for Neurology and Neurosurgery, London, United Kingdom; 2 Wellcome EPSRC Centre for Interventional and Surgical Sciences, University College London, London, United Kingdom; 3 School of Biomedical Engineering and Imaging Sciences, St Thomas’ Hospital, King's College London, London, United Kingdom; 4 Department of Clinical and Experimental Epilepsy, UCL Institute of Neurology, London, United Kingdom

**Keywords:** Surgery, Biopsy, Computer-assisted surgery, Automated

## Abstract

**BACKGROUND:**

Stereotactic brain biopsy is among the most common neurosurgical procedures. Planning an optimally safe surgical trajectory requires careful attention to a number of features including the following: (1) traversing the skull perpendicularly; (2) minimizing trajectory length; and (3) avoiding critical neurovascular structures.

**OBJECTIVE:**

To evaluate a platform, SurgiNav, for automated trajectory planning in stereotactic brain biopsy.

**METHODS:**

A prospectively maintained database was searched between February and August 2017 to identify all adult patients who underwent stereotactic brain biopsy and for whom postoperative imaging was available. In each case, the standard preoperative, T1-weighted, gadolinium-enhanced magnetic resonance imaging was used to generate a model of the cortex. A surgical trajectory was then generated using computer-assisted planning (CAP) , and metrics of the trajectory were compared to the trajectory of the previously implemented manual plan (MP).

**RESULTS:**

Fifteen consecutive patients were identified. Feasible trajectories were generated using CAP in all patients, and the mean angle determined using CAP was more perpendicular to the skull than using MP (10.0° vs 14.6° from orthogonal; *P* = .07), the mean trajectory length was shorter (38.5 vs 43.5 mm; *P* = .01), and the risk score was lower (0.27 vs 0.52; *P* = .03).

**CONCLUSION:**

CAP for stereotactic brain biopsy appears feasible and may be safer in selected cases.

**ABBREVIATIONS**
CAP: computer-assisted plan
CT: computed tomography
DBS: deep brain stimulation
MP: manual plan
MR: magnetic resonance
MRI: magnetic resonance imaging
SEEG: stereoelectroencephalography
STROBE: Strengthening the Reporting of Observational Studies in Epidemiology

Stereotactic brain biopsy is among the most common neurosurgical procedures. The principles of stereotactic surgery were introduced by Horsley and Clarke^1^ over a century ago to explore the primate brain, and brought into surgical practice by Spiegel et al.^2^ With the introduction of computed tomography (CT) in the 1970s and magnetic resonance imaging (MRI) in the 1980s, stereotactic biopsy has become increasingly widespread. Indications for stereotactic biopsy include brain lesions that are deep seated, present in eloquent areas, or located at multiple sites, for which there remains diagnostic uncertainty. Stereotactic biopsy is safe and effective in most cases, with a recent large series finding a diagnostic yield of 98.2%, a morbidity rate of 8.5%, and a mortality rate of 0.6%.^3^

Planning an optimal trajectory for stereotactic brain biopsy requires careful attention to a number of features, including the following: (1) traversing the skull perpendicularly; (2) minimizing trajectory length; and (3) avoiding critical neurovascular structures. In cases such as brainstem biopsy, which necessitates proximity to numerous critical neurovascular structures over a long length, planning a safe surgical trajectory can be particularly challenging and time-consuming.

Current commercially available software allows the surgeon to select the target lesion and an entry point, resulting in the generation of a manually planned trajectory. The surgeon may then review the trajectory in the axial, coronal, and sagittal planes and can also do so with a “probe's eye,” which offers a look-ahead view. A degree of trial and error is usually necessary to ensure an optimal trajectory.

Computer assistance may theoretically allow for safer and more straightforward surgical trajectory planning. Our group has previously reported the successful use of a software platform, EpiNav^TM^ (research software not commercially available; UCL, London, United Kingdom), in stereoelectroencephalography (SEEG) and laser interstitial thermal therapy.^4-7^ To this end, the aim of this study was to evaluate a related software platform, SurgiNav (research software not commercially available; UCL, London, United Kingdom), for computer-assisted planning in stereotactic brain biopsy.

## METHODS

A retrospective comparative pilot study design was adopted according to the IDEAL-D model (stage 0) for safe surgical innovation^8^; the SurgiNav software was used to generate computer-assisted plans (CAPs) retrospectively, and these were compared to the actually implemented manual plans (MPs) to determine feasibility and safety. The Strengthening the Reporting of Observational Studies in Epidemiology (STROBE) statement was used in the preparation of this section of the manuscript.^9^

The study was registered as a Service Evaluation study with the University College London Hospitals NHS Foundation Trust Clinical Audit Committee (NHNN2018050). Informed consent was not sought, as this was a retrospective study.

### Setting and Participants

The study was conducted at a university hospital that acts as a regional referral center for brain tumors.

All cases were recorded on a prospectively maintained database. The database was searched over a 6-mo period between February 1 and August 1, 2017, to identify all adult patients who had undergone stereotactic brain biopsy and for whom postoperative imaging was available, and, subsequently, the implemented MP was derived.

### Manual Plans

The MPs of surgical trajectories that were actually implemented were generated using a Stealth platform (Medtronic) by one of the senior neurosurgeons (N.K., A.W.M., A.M., and L.T.). In each case, the preoperative, volumetric, T1-weighted, gadolinium-enhanced magnetic resonance imaging (MRI) scan was used to identify the lesion(s). Entry and target points were placed using the axial, coronal, and sagittal planes, and the trajectory was checked using the probe's eye reconstruction.

### Computer-Assisted Plans

The CAPs of surgical trajectories were retrospectively generated using the SurgiNav platform by one of the neurosurgeons involved in the development of the platform (H.J.M. and V.V.). The technical aspects of the CAP algorithm have been described previously.^4-7^ In brief, the preoperative, volumetric, T1-weighted, gadolinium-enhanced MRI scan was used to create a skull model and perform whole-brain parcellation, which was then thresholded to create a cortical, gray matter, and sulcal model.^4-7^ A target lesion was segmented, and a maximum angle (30.0° from orthogonal) and length (100 mm) were defined by the surgeon. The CAP algorithm then calculated entry and target points and ranked these according to the risk score.

**FIGURE 1.**
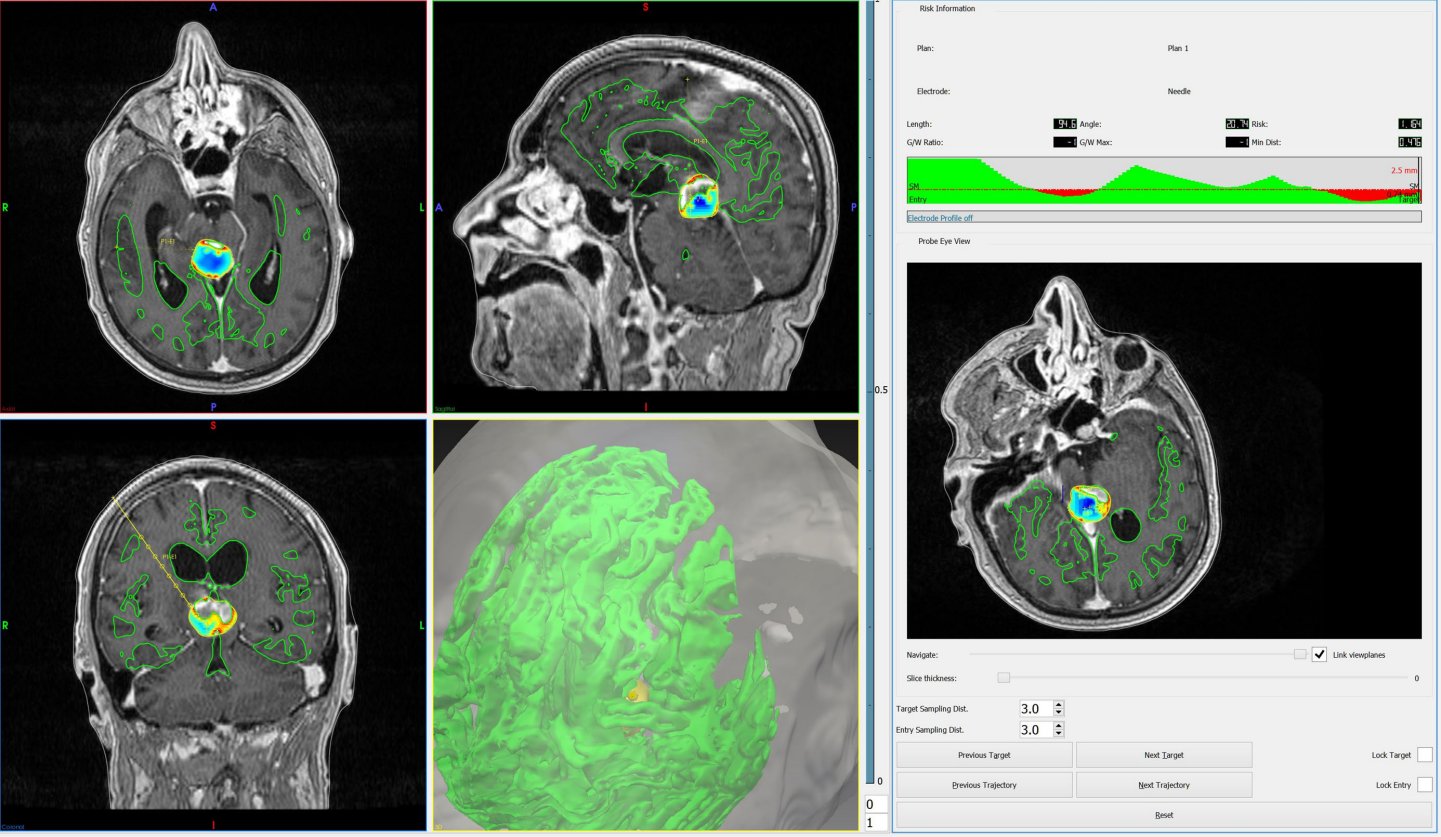
SurgiNav was used to generate a computer-assisted trajectory in a patient with a pineal region lesion. The left panel demonstrates the axial, coronal, and sagittal planes with the CAP trajectory in yellow. The right panel provides the trajectory metrics and allows the surgeon to cycle between CAP trajectories.

The risk score has previously been described as a function of the cumulative distance from vessels along the whole trajectory.^6^ In this study, we used sulci as the critical structure to avoid instead of the blood vessels because of the variable quality of the images available to segment the intracerebral vasculature. The risk score for a trajectory ranges from 0 (lowest risk) to 2 (highest risk): a risk score of 0 means the trajectory is always at least 10 mm away from the nearest sulcus; a risk score between 0 and 2 is the cumulative sum of how close the trajectory is to the nearest sulcus; and a risk score of 2 means the trajectory conflicts with a sulcus. The 5 trajectories with the lowest risk were reviewed by the neurosurgeon, and the most feasible one was chosen (Figure 1); this methodology has been adopted in previous studies to account for surgeon preference and to improve the feasibility of CAP trajectories.^5^

### Outcomes

Data were collected on the metrics of CAP and MP trajectories, diagnostic yield, morbidity, and mortality rates. Specimens were sent for histopathological analysis and considered positive if they resulted in a diagnosis. Immediate surgical complications were recorded according to the Clavien-Dindo classification.^10^,^11^

The primary outcomes of this comparative study were as follows: (1) trajectory angle from orthogonal; (2) trajectory length; and (3) risk score.

### Study Size and Statistical Methods

No formal power calculation was performed, as data on the primary outcomes were not available. Instead, the sample size was determined through adopting a constraint-based pragmatic approach and based on previous related studies.^12^ We considered a minimum of 12 patients in each group sufficient for a meaningful analysis in this pilot study, and it was estimated that this would be achieved over a 6-mo period.

Data were analyzed using SPSS v 20.0 (IBM, Chicago, Illinois). We have previously shown that trajectory metrics are normally distributed.^5^ The mean and standard deviations were calculated. Data were compared using the paired *t*-test, with a value of *P* < .05 considered statistically significant.

## RESULTS

### Participants and Descriptive Data

Fifteen consecutive patients were identified who had undergone stereotactic brain biopsy using MP surgical trajectories between February 1 and August 1, 2017, and for whom postoperative imaging was available. The patient demographics are detailed in Table 1. Their median age was 62 yr (range: 18-78 yr), and the male-to-female ratio was 4:1. Brain lesions were most commonly located in the frontal and parietal regions (8/15).

**TABLE 1.** Patient Demographics and Pathology

Case	Age (yr)	Sex (M/F)	Location	Pathology
1	70	F	Left parietal	GBM
2	62	F	Right parietal	GBM
3	66	M	Left frontal	GBM
4	78	M	Left frontal	GBM
5	62	M	Right frontal	GBM
6	54	M	Pineal region	GBM
7	72	M	Right occipital	GBM
8	72	M	Corpus callosum	GBM
9	56	M	Right parietal	GBM
10	44	F	Left frontal	GBM
11	64	M	Left temporal	GBM
12	46	M	Corpus callosum	Multifocal germinoma
13	18	M	Left temporal	Pilocytic astrocytoma
14	60	M	Left thalamic	GBM
15	57	M	Right parietal	GBM

M = male, F = female, GBM = glioblastoma multiforme.

### Outcome Data and Main Results

All patients had a diagnostic biopsy, and the patient pathologies are detailed in Table 1. The most common pathology was glioblastoma multiforme (13/15). There were no immediate surgical complications. The median length of postoperative inpatient stay was 4 d (range: 1-38 d).

The primary outcomes are detailed in Table 2. Feasible trajectories were generated using CAP in all patients. In case 6, the target lesion was located within the pineal region and the entry region was constrained to the right frontal lobe.

**TABLE 2.** Trajectory Angle From Orthogonal (°), Trajectory Length (mm), and Risk Score in MPs and CAPs

	MP			CAP		
Case	Angle (°)	Length (mm)	Risk	Angle (°)	Length (mm)	Risk
1	9.6	35.0	0.18	7.8	35.5	0.02
2	15.4	49.6	0.00	0.5	44.8	0.00
3	16.3	42.4	1.21	10.6	49.4	1.03
4	25.5	30.1	0.00	4.1	17.0	0.00
5	15.0	39.4	0.00	9.0	27.0	0.00
6	18.9	95.4	1.09	20.7	94.6	1.16
7	30.1	17.5	0.00	9.3	16.6	0.00
8	2.2	46.8	1.16	9.2	41.2	0.00
9	11.0	29.1	0.00	4.2	14.1	0.00
10	18.2	28.8	0.00	14.8	15.1	0.00
11	5.4	39.9	1.03	14.4	39.3	0.37
12	20.9	55.6	1.15	14.6	49.9	0.13
13	9.0	47.3	0.00	1.2	45.1	0.00
14	7.9	64.1	1.03	16.5	57.6	1.04
15	14.2	31.6	1.00	12.7	31.0	0.32

CAP = computer-assisted plan, MP = manual plan.

**FIGURE 2.**
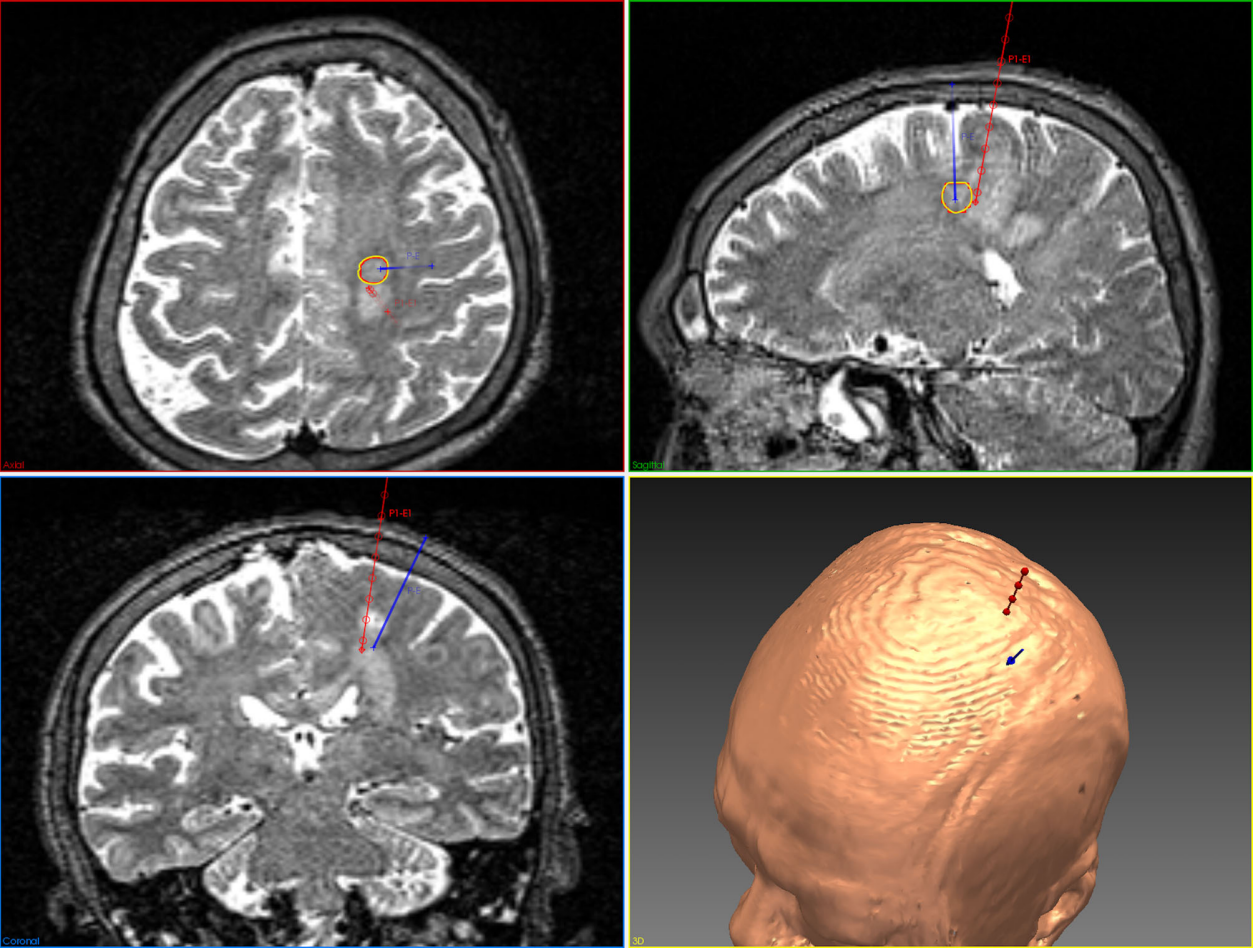
SurgiNav was used to compare CAP (blue) and MP (red) trajectories in a patient with a left frontal lesion. Note that whereas a T1-weighted, gadolinium-enhanced MRI was used to generate a model of the cortex, a T2-weighted MRI has been used in this figure to better illustrate the lesion.

**FIGURE 3.**
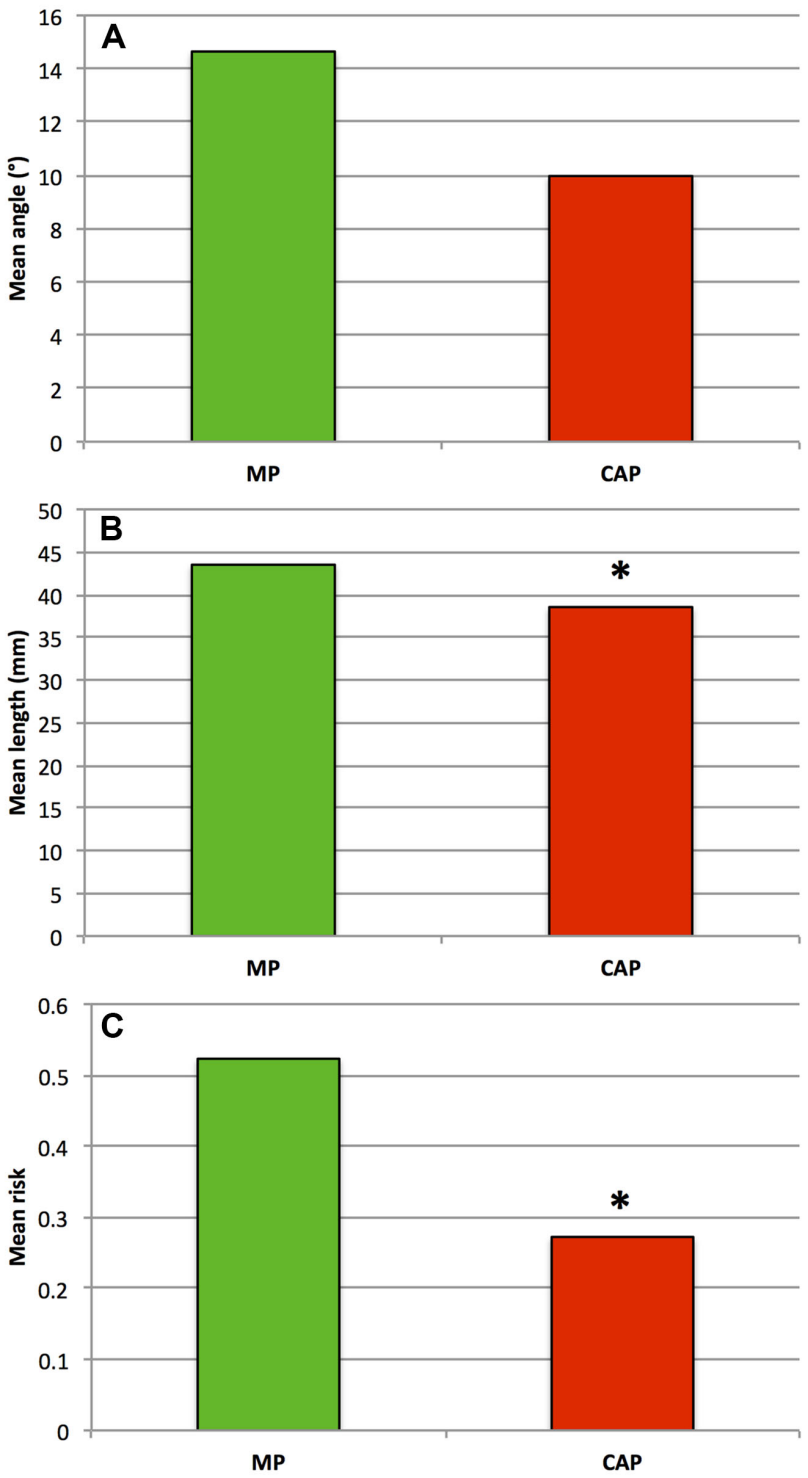
Graphs comparing MPs and CAPs: **A**, mean trajectory angle from orthogonal (°); **B**, mean trajectory length (mm); and **C**, mean risk score. *P < .05.

An illustrative case comparing CAP and MP trajectories is shown in Figure 2. The mean angle using CAP was more perpendicular to the skull than using MP (10.0° vs 14.6° from orthogonal; *P* = .07), the mean trajectory length was shorter (38.5 vs 43.5 mm; *P* = .01), and the risk score was lower (0.27 vs 0.52; *P* = .03) (Figure 3).

## DISCUSSION

### Principal Findings

We found that CAP for stereotactic brain biopsy appears feasible and may be safer than MP in selected cases. The SurgiNav platform was able to generate feasible trajectories in all cases, the CAP trajectories were significantly shorter, and the risk scores were lower than those of MP trajectories. These findings are promising and merit stage 1 and 2 clinical trials in accordance with the IDEAL-D model for safe surgical innovation.^8^

### Comparison With Other Studies

This is the first study that describes CAP for surgical trajectory planning in stereotactic brain biopsy. CAP has been used in neurosurgery for over 30 yr,^13^ and the successful use of CAP for surgical trajectory planning has been described for deep brain stimulation (DBS),^12^,^14^ SEEG,^4-6^,^15^ and laser trajectory planning.^7^

Beriault et al^12^ developed a platform for CAP in DBS. Their trajectory planning algorithm analyzed every trajectory connecting the ipsilateral frontal lobe to a surgeon-defined target point using a 2-pass technique. In the first pass, trajectories that traversed the ventricles or were too close to sulci were eliminated. In the second pass, the remaining trajectories were ranked according to their distance from all critical structures. In a retrospective comparative study of 14 patients who had undergone DBS for Parkinson disease, feasible trajectories were generated in all cases, and the CAP trajectories appeared to have favorable risk metrics compared to the implemented MP trajectories, although no statistical analysis was performed.

De Momi et al^15^ developed a platform for CAP in SEEG. Their trajectory planning algorithm analyzed every trajectory connecting approximate surgeon-defined entry and target points and ranked these according to their distance from critical structures and the drilling angle. In a retrospective analysis of 26 electrodes in 3 patients undergoing SEEG, a feasible trajectory was generated in 86% of cases, and the CAP trajectories resulted in a significantly greater distance from vessels compared to that of the implemented MP trajectories.

Our group has previously reported the successful use of a software platform, EpiNav^TM^, in SEEG.^4-6^ In an initial study of 166 electrodes in 18 patients undergoing SEEG, a feasible trajectory was generated in 79% of cases, and the CAP trajectories resulted in a significantly reduced risk score compared to that of the implemented MP trajectories.^4^ In a subsequent study of 116 electrodes in 13 patients undergoing SEEG, we improved the algorithm.^5^ Rather than a single surgeon-defined target point, an entire anatomical structure could be selected based on whole-brain parcellation, allowing the algorithm to select the safest target within the anatomical structure of interest.

EpiNav^TM^ has most recently been applied to laser interstitial thermal therapy. In a retrospective study of 25 patients who underwent laser interstitial thermal therapy for mesial temporal lobe epilepsy, a feasible trajectory was generated in all cases. The mean risk score obtained using CAP was lower than that obtained using MP, and the trajectory length was shorter. Furthermore, CAP trajectories would have resulted in a greater ablation of the amygdala and amygdalohippocampal complex.

In this study, the application of SurgiNav to stereotactic brain biopsy had constraints when compared to the previous use of EpiNav^TM^ for SEEG and laser interstitial thermal therapy. Patients undergoing surgery for epilepsy routinely undergo extensive MRI, including MR angiography and venography, whereas patients undergoing stereotactic brain biopsy in our institution routinely undergo volumetric, T1-weighted, gadolinium-enhanced MRI alone so that a reliable segmentation of blood vessels is not possible. In consequence, we used sulci as the critical structure to avoid instead of the blood vessels themselves.

Several studies have described multimodal imaging in stereotactic brain biopsy, and it has been suggested that selecting targets within regions of high relative cerebral blood flow or specific signatures on MR spectroscopy may improve biopsy yield.^16^,^17^ In the future, we will combine the use of such multimodal imaging with SurgiNav to improve the safety and efficacy of stereotactic brain biopsy.

### Limitations

As noted, we used sulci as the critical structure to avoid instead of blood vessels themselves because of the variable quality of the vascular imaging. The risk of hemorrhage is significantly greater when trajectories traverse sulci.^18^ As the risk score was the primary optimization criterion for CAP, using this score to compare CAP and MP trajectories inevitably resulted in bias toward CAP. In future studies, we will assess how well the risk score predicts complications in patients undergoing stereotactic brain biopsy, but, at present, we are unable to extrapolate the relative risk reduction of hemorrhage.

The time required to generate CAP and MP trajectories was not recorded in this retrospective study. Although SurgiNav was able to calculate entry and target points in a few minutes, the prerequisite whole-brain parcellation took up to an hour, albeit on a workstation without surgeon intervention over this time. This represents a potential drawback to the clinical use of SurgiNav in stereotactic brain biopsy, which, unlike SEEG or laser interstitial thermal therapy, is often undertaken as a matter of clinical urgency. We will optimize the whole-brain parcellation algorithm, and take advantage of the increasing computing power, to reduce the time taken.

The small and retrospective nature of this study has the potential for bias. Nonetheless, the findings encourage larger prospective clinical studies.

## CONCLUSION

CAP for stereotactic brain biopsy appears feasible and may be safer in selected cases. The findings of this retrospective comparative pilot study merit further development of the SurgiNav platform and stage 1 and 2 clinical trials in accordance with the IDEAL-D model for safe surgical innovation.

### Disclosures

This publication represents, in part, an independent research commissioned by the Health Innovation Challenge Fund (HICF-T4-275, WT097914, and WT106882), a parallel funding partnership between the Wellcome Trust and the Department of Health, and the National Institute for Health Research University College London Hospitals Biomedical Research Centre (NIHR BRC UCLH/UCL High Impact Initiative). The authors have no personal, financial, or institutional interest in any of the drugs, materials, or devices described in this article.

## Notes

Interim findings of this work were shared as an oral presentation at the meeting of the Society of British Neurological Surgeons in London, United Kingdom, on September 19, 2018.
